# Tumor-infiltrating lymphocytes and immune-related adverse events in advanced melanoma

**DOI:** 10.1016/j.iotech.2024.100714

**Published:** 2024-06-12

**Authors:** I.A.J. van Duin, M. Schuiveling, L.S. ter Maat, M. Veta, M.J.M. van Eijs, R.J. Verheijden, F.W.P.J. van den Berkmortel, M.J. Boers-Sonderen, G.A.P. Hospers, M. Labots, J.W.B. de Groot, E. Kapiteijn, D. Piersma, G. Vreugdenhil, H. Westgeest, A.M.R. Schrader, P.J. van Diest, W.A.M. Blokx, K.P.M. Suijkerbuijk

**Affiliations:** 1 Department of Medical Oncology, University Medical Center Utrecht, Utrecht University, Utrecht; 2 Image Sciences Institute, University Medical Center Utrecht, Utrecht University, Utrecht; 3 Medical Image Analysis, Department of Biomedical Engineering, Eindhoven University of Technology, Eindhoven; 4 Center for Translational Immunology, University Medical Center Utrecht, Utrecht University, Utrecht; 5 Julius Centre for Health Sciences and Primary Care, University Medical Center Utrecht, Utrecht University, Utrecht; 6 Department of Medical Oncology, Zuyderland Medical Centre, Sittard-Geleen; 7 Department of Medical Oncology, Radboud University Medical Centre, Nijmegen; 8 Department of Medical Oncology, University Medical Centre Groningen, University of Groningen, Groningen; 9 Department of Medical Oncology, Amsterdam UMC, VU University Medical Center, Cancer Center Amsterdam, Amsterdam; 10 Isala Oncology Center, Isala, Zwolle; 11 Department of Medical Oncology, Leiden University Medical Centre, Leiden; 12 Department of Internal Medicine, Medisch Spectrum Twente, Enschede; 13 Department of Internal Medicine, Maxima Medical Centre, Eindhoven; 14 Department of Internal Medicine, Amphia Hospital, Breda; 15 Department of Pathology, Leiden University Medical Centre, Leiden; 16 Department of Pathology, University Medical Center Utrecht, Utrecht University, Utrecht, The Netherlands

**Keywords:** melanoma, immunotherapy, immune-related adverse events, pathology, tumor-infiltrating lymphocytes

## Abstract

**Background:**

The predictive value of tumor-infiltrating lymphocytes (TILs) in immune-related adverse event (irAE) development remains unknown, although an association between tumor immunogenicity and irAEs has been suggested. We investigated the association between TIL abundance in pretreatment primary and metastasis specimens and the subsequent development of severe irAEs.

**Patients and methods:**

We retrospectively identified patients with advanced cutaneous melanoma who received first-line anti-programmed cell death protein 1 (PD-1) with or without anti-cytotoxic T-lymphocyte associated protein 4 (anti-CTLA-4) from 10 hospitals in the Netherlands. TILs were scored on representative hematoxylin and eosin (H&E) stains of the primary melanoma and pretreatment melanoma metastasis as ‘absent’, ‘nonbrisk’, or ‘brisk’. A univariable logistic regression analysis was carried out to assess the association between the TIL scores and the development of severe irAEs. Fine and Gray subdistribution hazard models were used to estimate the cumulative incidence of severe irAEs.

**Results:**

Of the 1346 eligible patients, 536 patients had primary melanoma specimens available, and 613 patients had metastasis specimens available. Severe irAEs occurred in 15% of anti-PD-1-treated patients and 49% of anti-PD-1 + anti-CTLA-4-treated patients. The presence of TILs was not associated with the occurrence of grade ≥3 irAEs in primary melanoma specimens (*P* = 0.70) nor pretreatment metastasis specimens (*P* = 0.91). In the univariable analysis, patients with brisk TILs did not have a higher chance of developing severe irAEs compared with patients with absent TILs, for both primary specimen (odds ratio 1.15, 95% confidence interval 0.60-2.18) and metastasis specimen (odds ratio 0.77, 95% confidence interval 0.37-1.59). There was also no significant difference in the lifetime risk or timing of the development of severe irAEs in patients with TILs present compared with patients with TILs absent.

**Conclusion:**

There was no association between the TIL scores on H&E-stained slides from the primary melanoma or pretreatment metastasis and the development of grade 3 or higher irAEs. Additionally, no correlation was found between the presence of TILs and the timing of irAEs.

## Highlights

• Examining the role of TILs in severe irAE development in immune checkpoint inhibitor treated patients.
• Five hundred and thirty-six patients had primary melanoma specimens, and 613 patients had pretreatment metastasis specimens available.
• TILs were scored on H&E-stained slides as ‘absent’, ‘nonbrisk’, or ‘brisk’ in primary melanoma and metastasis specimens.
• No significant association was found between TIL abundance and the occurrence of grade ≥3 irAEs.
• The presence of TILs did not correlate with the development or timing of severe irAEs.

## Introduction

The prognosis for patients with advanced cutaneous melanoma has greatly improved since the introduction of immune checkpoint inhibition (ICI). In real-world data, the 4-year overall survival probability for ipilimumab- and nivolumab-treated patients surpasses 40%.^1^ Nonetheless, half of patients do not respond to this therapy. In addition, patients are prone to experience potentially severe immune-related adverse events (irAEs).^2^

The grading of irAEs is based on the Common Terminology Criteria for Adverse Events (CTCAE) version 4.03.^3^ Severe irAEs (grade ≥3) are observed in ∼15% of anti-programmed cell death protein 1 (PD-1)-treated patients, and in 60% of patients treated with combination therapy.^4^^,^^5^ IrAEs can be life-threatening and frequently lead to ICI discontinuation, treatment with immune-modulating agents, and a lifelong need for hormone suppletion.^6^ The incidence of irAEs varies primarily based on the ICI regimen, with slight variations in the occurrence of irAE subtypes observed between tumor types. For example, gastrointestinal and skin irAEs were observed more frequently in melanoma compared with other tumors.^7^

Although there is evidence linking irAEs to the immunological antitumor effects of ICI, the precise mechanisms underlying checkpoint inhibitor toxicity are complex and not fully understood.^8^ Many factors have been associated with the development of irAEs, including medical history, medication use, (tumor-specific) ICI regimens and dosing, and microbiome composition.^9^^,^^10^ Prior research has been focusing on the search for biomarkers for irAEs in melanoma.^11^ However, the task of effectively screening and identifying patients who are prone to irAEs remains challenging.^12^

Tumor-specific differences in irAE patterns might be explained by differences in the tumor microenvironment and shared antigens between specific tumors and healthy tissue. For example, a recent study found that patients with acral melanoma were less likely to develop cutaneous irAEs compared with patients with nonacral cutaneous melanoma.^13^ This variation in irAE incidence might be attributed to the disparity in immunogenicity observed between the two subtypes.^14^ To add to that, a positive association between irAEs and tumor mutational burden, and thus tumor immunogenicity, has been shown.^15^

While tumor-infiltrating lymphocytes (TILs) are known to be associated with improved long-term survival and better responses to ICI,^16^ their specific role in irAE development remains unclear. Notably, a correlation exists between ICI response and the development of irAEs.^17^

In this study, we explored the relationship between the TIL scores on hematoxylin and eosin (H&E)-stained slides from both pretreatment primary and metastasis specimens of patients with advanced melanoma undergoing ICI treatment and the development of grade ≥3 irAEs. First, we assessed the relationship between the presence of TILs and the development of severe irAEs. Second, we investigated the timing of the development of severe irAEs with respect to the presence of TILs.

## Methods

### Patients and outcomes

For this study, we retrospectively identified patients with advanced cutaneous melanoma from 10 centers in the Netherlands. Clinical data were extracted from prospectively collected high-quality registry data.^18^ Inclusion criteria were age >18 years and treatment with first-line anti-PD-1 monotherapy or anti-PD-1 + anti-cytotoxic T-lymphocyte associated protein 4 (anti-CTLA-4; ipilimumab and nivolumab) for irresectable stage IIIC or stage IV cutaneous melanoma after 1 January 2016.

The stage of disease was determined based on the 8th edition of the American Joint Committee on Cancer (AJCC) Melanoma Staging System.^19^

In the collected data, only severe irAEs (defined as CTCAE version 4.03 grade ≥3 toxicity) were reported.^3^ The outcome of this study was, therefore, defined as the occurrence of grade ≥3 toxicity during ICI treatment. Patients with a missing outcome were excluded from the analysis.

### Sample selection and assessment of TILs

If available, a single H&E-stained slide was chosen from the primary melanoma and the metastatic site for each patient. In cases with multiple primary melanomas, the melanoma with the highest Breslow thickness or the most suspicious location in terms of regional lymph node involvement was picked. When multiple specimens from metastatic sites were present, the most recent slide before treatment initiation was selected. The selected slides were scanned with a NanoZoomer-XR C12000-21/-22 (Hamamatsu Photonics, Hamamatsu, Shizuoka, Japan) at ×40 magnification with a resolution of 0.22 μm per pixel. TIL scoring was conducted by authors IAJD and MS and supervised by experienced pathologists (PJvD and WAMB) who were all unaware of patients’ outcomes at the time of scoring. TILs were scored as ‘absent’, ‘nonbrisk’, or ‘brisk’, according to the scoring system proposed by Clark et al.^20^ (examples are shown in Supplementary Figure S1, available at https://doi.org/10.1016/j.iotech.2024.100714). In addition, the Melanoma Institute Australia (MIA) scoring and stromal scoring system (stromal score) were used, as comprehensively described in Supplementary Tables S1-S3, available at https://doi.org/10.1016/j.iotech.2024.100714. In a minority of cases, the stromal score could not be assessed because of the lack of tumoral stroma (e.g. when the slide contained only a few tumor cells).

### Statistical analysis

To describe the study population, we used medians and interquartile intervals for continuous variables, and percentages and frequencies for categorical variables. Interobserver agreement was assessed with Cohen’s kappa for the Clark score and MIA score. Intraclass correlation and Bland–Altman analysis were used for the assessment of interobserver agreement in the stromal score. We used chi-square tests to evaluate the association between categorical TIL scores (Clark score and MIA score) and outcomes. Associations between stromal score and outcomes were assessed using Mann–Whitney *U* tests as data were not normally distributed. Univariable logistic regression analyses were carried out to assess the association between the TIL scores and the occurrence of severe irAEs, specifically severe colitis. As most irAEs (>93%) occur within 1 year of treatment initiation,^21^^,^^22^ patients with <1-year follow-up were excluded from logistic regression analyses if they were alive at the last follow-up to prevent misclassification of irAE outcome, as an irAE may have occurred after the last follow-up.

Fine and Gray subdistribution hazard models were used to estimate the cumulative incidence of severe irAEs, accounting for the competing risk of death. This also provides insights into possible differences in the time to irAE onset. Overall, 5% of cases had missing data, so we carried out a complete case analysis. Analyses were conducted using R statistical software (version 4.2.2; R Foundation, Vienna, Austria).

The study design was reviewed by the Medical Ethics Committee and not considered subject to the Medical Research Involving Human Subjects Act in compliance with Dutch regulations; informed consent was waived.

## Results

**Figure 1:**
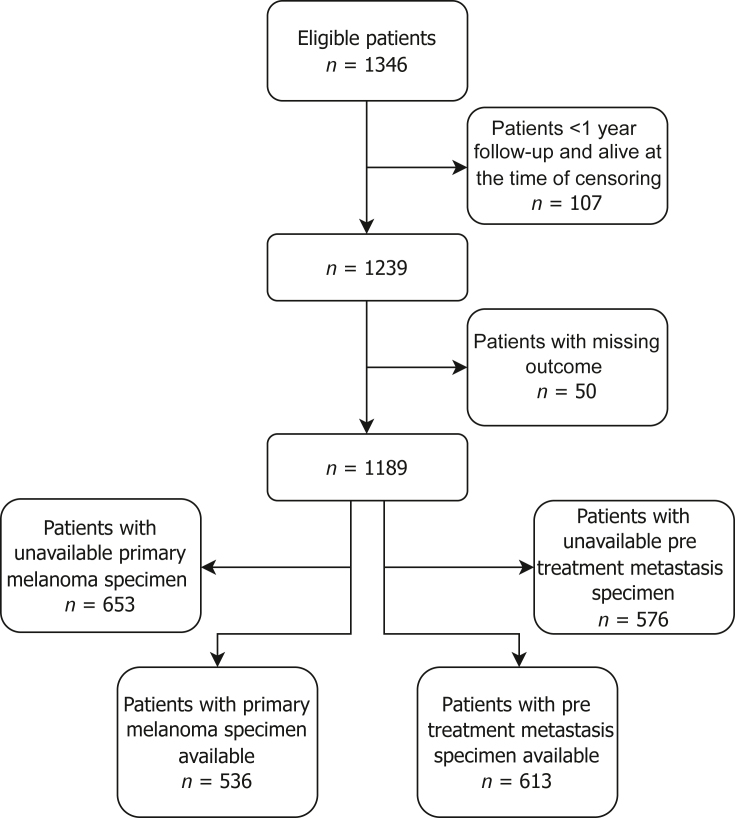
Flowchart of the study population.

Of the 1346 eligible patients, 536 had a primary melanoma specimen available, and 613 had a metastasis specimen available (Figure 1). Baseline characteristics of the two groups were comparable (Table 1). More patients in the primary melanoma group had TILs present in their samples compared with the metastatic melanoma group.

**Table 1:** Baseline characteristics of 536 patients with advanced cutaneous melanoma treated with anti-PD-1-based therapy, with primary melanoma specimen available, and 613 patients with advanced cutaneous melanoma treated with anti-PD-1 or ipilimumab and nivolumab with pretreatment metastasis specimen available

Characteristics	Primary specimen available (*n* = 536)	Metastasis specimen available (*n* = 613)
Age (years)		
Median (interquartile interval)	68.0 (58.0-75.0)	66.0 (57.0-74.0)
Sex, *n* (%)		
Female	186 (34.7)	207 (33.8)
Male	350 (65.3)	406 (66.2)
WHO performance status		
WHO 0, *n* (%)	245 (47.4)	267 (45.2)
WHO 1, *n* (%)	227 (43.9)	279 (47.2)
WHO 2-4, *n* (%)	45 (8.7)	45 (7.6)
Missing, *n*	19	22
Stage of disease		
Unresectable IIIC, *n* (%)	39 (7.6)	45 (7.6)
M1a, *n* (%)	32 (6.2)	38 (6.5)
M1b, *n* (%)	76 (14.8)	78 (13.2)
M1c, *n* (%)	249 (48.4)	268 (45.5)
M1d, *n* (%)	118 (23.0)	160 (27.2)
Missing, *n*	22	24
*BRAF* V600 mutation		
Wildtype, *n* (%)	350 (70.1)	377 (65.8)
Mutant, *n* (%)	149 (29.9)	196 (34.2)
Missing, *n*	37	40
*NRAS* mutation		
Wild type, *n* (%)	279 (60.7)	312 (62.7)
Mutant, *n* (%)	181 (39.3)	186 (37.3)
Missing, *n*	76	115
LDH levels		
Not elevated, *n* (%)	343 (64.6)	391 (64.5)
1-2× ULN, *n* (%)	140 (26.4)	167 (27.6)
>2× ULN, *n* (%)	48 (9.0)	48 (7.9)
Missing, *n*	5	7
Type of systemic therapy, *n* (%)		
Anti-PD-1	346 (64.6)	382 (62.3)
Ipilimumab and nivolumab	190 (35.4)	231 (37.7)
Presence of tumor-infiltrating lymphocytes, *n* (%)		
Absent	129 (24.1)	322 (52.5)
Nonbrisk	323 (60.3)	229 (37.4)
Brisk	84 (15.7)	62 (10.1)
Severe toxicity (grade ≥3), *n* (%)		
No	401 (74.8)	431 (70.3)
Yes	135 (25.2)	182 (29.7)

LDH, lactate dehydrogenase; PD-1, programmed cell death protein 1; ULN, upper limit normal; WHO, World Health Organization.

Of the 536 primary melanoma specimens available, 84 (15.7%) were scored as having brisk TILs, 323 (60.3%) as having nonbrisk TILs, and 129 (24.1%) as having no TILs. In metastatic samples, TILs were frequently absent (*n* = 322, 52.5%), with brisk TILs only observed in 62 (10.1%) and nonbrisk TILs in 229 (37.4%) patients. No association was found between TILs and *BRAF* or *NRAS* mutational status (Supplementary Figure S2, available at https://doi.org/10.1016/j.iotech.2024.100714). The frequencies of all three TIL scores in the two patient groups are shown in Supplementary Table S4, available at https://doi.org/10.1016/j.iotech.2024.100714. Patient characteristics of included versus excluded patients are presented in Supplementary Table S5, available at https://doi.org/10.1016/j.iotech.2024.100714. The median follow-up time was 36 months. Overall, the median progression-free survival was 8 months and the median overall survival was 28 months.

### Association between severe irAEs and TIL score

Overall, ∼27% of patients experienced grade ≥3 irAEs during their treatment. In patients who were treated with anti-PD-1 monotherapy, grade ≥3 irAEs occurred in 15% of patients. Among patients treated with ipilimumab or nivolumab, 49% experienced grade ≥3 irAEs.

**Figure 2:**
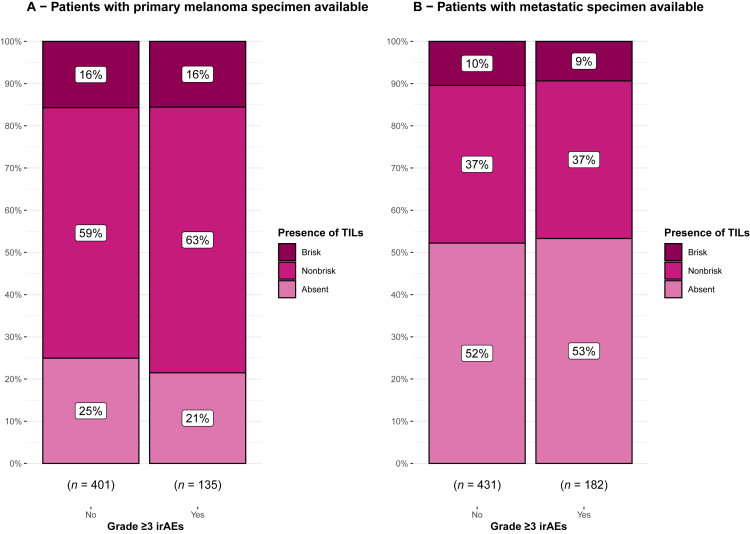
**Stacked bar chart of comparison of occurrence of grade ≥3 immune-related adverse events (irAEs) in patients categorized by their tumor-infiltrating lymphocyte (TIL) score (absent, nonbrisk, or brisk).** (A) TILs scored in the primary specimen (*P* = 0.70). (B) TILs scored in metastasis (*P* = 0.91).

The presence of TILs was not associated with the occurrence of grade ≥3 irAEs (Figure 2) in primary melanoma specimens (*P* = 0.70) nor pretreatment metastasis specimens (*P* = 0.91). This observation was consistent across all three TIL scoring systems (Supplementary Figure S3, available at https://doi.org/10.1016/j.iotech.2024.100714). In the univariable analysis, the TIL score was also not associated with the occurrence of severe irAEs (Table 2). Furthermore, we found no association between the accumulation of toxicity in different organs and TIL scoring (Supplementary Table S6, available at https://doi.org/10.1016/j.iotech.2024.100714). When stratifying for the type of treatment, there was also no association between the TIL scores and severe toxicity (Supplementary Figure S4, available at https://doi.org/10.1016/j.iotech.2024.100714). In an additional analysis with the development of grade ≥3 colitis as an outcome, no significant association was found between the presence of TILs and colitis (Supplementary Table S7, available at https://doi.org/10.1016/j.iotech.2024.100714).

**Table 2:** Univariable logistic regression analysis of TIL presence and the occurrence of severe irAEs in patients with ICI-treated advanced melanoma

TILs scored in primary melanoma specimen			
Presence of TILs	Univariable analysis		
	OR	95% CI	*P* value
Absent	—		
Nonbrisk	1.23	0.77-2.02	0.40
Brisk	1.15	0.60-2.18	0.67

**TILs scored in pretreatment metastasis specimen**			
**Presence of TILs**	**OR**	**95% CI**	***P* value**
Absent	—		
Nonbrisk	0.98	0.68-1.42	0.91
Brisk	0.88	0.47-1.58	0.67

CI, confidence interval; ICI, immune checkpoint inhibition; irAE, immune-related adverse event; OR, odds ratio; TIL, tumor-infiltrating lymphocyte.

### TILs and the timing of toxicity

**Figure 3:**
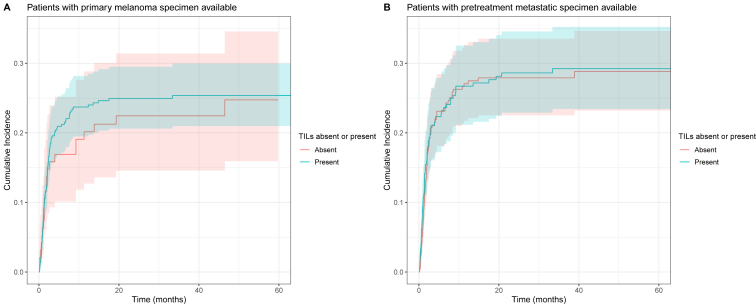
**Cumulative incidence of development of grade ≥3 immune-related adverse events (irAEs) in patients with advanced melanoma treated with immune checkpoint inhibition (ICI), according to tumor-infiltrating lymphocyte (TIL) presence (absent or present).** 95% confidence bands are shown. (A) TILs scored in the primary specimen. (B) TILs scored in metastasis.

Lastly, we investigated the relationship between the presence of TILs and the timing of the development of severe irAEs. For three centers, we did not have time-to-toxicity data available and thus had to exclude 328 patients from our analysis. A flowchart of the studied patients is shown in Supplementary Figure S5, available at https://doi.org/10.1016/j.iotech.2024.100714. The TIL score was dichotomized as ‘absent’ or ‘present’. There was no significant difference in the lifetime risk or timing of the development of severe irAEs in patients with TILs present compared with patients with TILs absent, both in the cohort having primary specimen available (subdistribution hazard ratio 1.10, 95% confidence interval 0.69-1.74) and in the cohort having metastasis specimen available (subdistribution hazard ratio 1.03, 95% confidence interval 0.74-1.44). The cumulative incidence of the development of severe irAEs is shown in Figure 3.

## Discussion

Our study found no association between TILs on H&E-stained slides and the development of grade ≥3 irAEs in patients with advanced cutaneous melanoma undergoing ICI treatment. This finding was consistent for TIL scores in both the primary melanoma and pretreatment metastasis specimens, and when examining immune checkpoint inhibitor colitis specifically. Furthermore, we found no correlation between the presence of TILs and the timing of irAEs.

Stephens et al.^23^ investigated the association between TILs in primary melanomas as mentioned in pathology reports and the development of irAEs in 210 patients. The authors found no associations between TIL status and the development of irAEs. We are the first to assess TILs on H&E-stained slides on both primary melanoma and pretreatment metastasis specimens in relation to the development of grade ≥3 irAEs. Our study, with a larger cohort also including pretreatment metastasis and in which the TIL status was scored directly from slides instead of from reports, corroborates these results.

A recent study by Kerepesi et al.^24^ in 378 patients with non-small-cell lung carcinoma showed an increased proportion of TILs in ICI-treated patients with irAEs. However, comparing these results with our own findings is challenging because of the difference in tumor type and their inclusion of all grades of irAEs. Furthermore, the authors solely quantified TILs, whereas we used a qualitative grading system (absent, brisk, and nonbrisk) to evaluate TILs.

ICI treatment often results in irAEs, posing significant clinical challenges. The mechanisms behind these toxicities are complex and not fully understood.^25^ Besides, response and toxicity to ICI are correlated, complicating the identification of distinct biomarkers for irAEs. Recently, Prokhnevska et al.^26^ showed that CD8^+^ T-cell activation in response to cancer might consist of two stages of tumor-specific CD8^+^ T-cell activation. After the initial priming of T cells in lymph nodes, full effector differentiation takes place within the tumor itself. This is corroborated by recent experimental data on T-cell tolerance in peripheral tissue demonstrating that PD-1 prevents invading of T cells that were already primed against neoantigens in healthy tissue from achieving full effector differentiation.^27^ Along these lines, one could hypothesize that the TIL pattern is associated with ICI response but not with irAEs because brisk TILs primarily reflect an efficient environment for final effector differentiation within the tumor, which occurs separately in irAE-affected tissue.

Our study has several strengths, including the large and multicenter character cohort. Our cohort consists of patients from 10 hospitals, both academic and nonacademic, and is therefore representative of a population of ICI-treated patients with advanced melanoma.

There are also limitations. First, we lacked data on lower grades of toxicity. For example, cutaneous irAEs, which are more common in melanoma, are seldom classified as severe. Second, a substantial number of patients were excluded because of the unavailability of their pathology specimens. We think, however, that our data resemble the general population well because the baseline characteristics of included versus excluded patients were comparable.

In conclusion, we found no evidence that the presence of TILs on H&E-stained slides in both the primary melanoma and the pretreatment metastatic sample is associated with the development or timing of grade ≥3 irAEs. This offers hope for disentangling ICI efficacy and toxicity, for example, by targeting T-cell or microenvironment-specific parameters, both in the tumor microenvironment and in the irAE microenvironment.
