# Supplementary material for: Tumor-infiltrating lymphocytes and immune-related adverse events in advanced melanoma
Source: Immunooncol Technol. 2024 Jun 12;24:100714. doi: 10.1016/j.iotech.2024.100714 (PMC11262179; doi:10.1016/j.iotech.2024.100714)
Supplement: Supplementary data [file mmc1.docx]

**Supplementary materials**

Supplementary table 1. TILs scoring system as described by Clark *et al*, further elaborated by Clemente *et al*.^1,2^

| **TIL grade** | **Explanation** |
| --- | --- |
| Absent | No TILs in tumor tissue |
| Non-brisk - localized | Patchy multifocal lymphocytic infiltrate or mildly diffuse, but overall little TILs throughout the tumor |
| Brisk | TILs infiltrate the entire base of the tumor and/or diffusely infiltrate the entire invasive component of the tumor |

Supplementary table 2. TIL scoring system as described by the Melanoma Institute Australia (MIA).^3^

| **TIL grade** | **Explanation** |
| --- | --- |
| 0 | Absent |
| 1 | Mild infiltrate of lymphocytes focally interspersed among the melanoma cells. |
| 2 | Dense multifocal infiltrate of lymphocytes interspersed among the melanoma cells. |
| 3 | Dense infiltrate of lymphocytes illustrated in the figure was present diffusely throughout the tumor. |

Supplementary table 3. Stepwise approach of assessing TILs in the tumoral stroma, as described by Hendry.^4^

| Step 1 | Define the tumor area. Exclude areas with immune infiltrate outside tumor border. |
| --- | --- |
| Step 2 | Distinguish between intratumoral TILs and tumoral stromal TIL’s |
| Step 3 | Identify the type of inflammatory infiltrate, excluding granulocytes and necrosis. |
| Step 4 | Assess the percentage of TILs. |
| In our analysis, The percentage can range from 0 to 100%, in increments of 5%. | |

Supplementary table 4. The frequencies of all three TILs scores in the two patient groups IQI: interquartile interval

|  | **Patients with primary specimen available** | **Patients wit pre-treatment metastatic specimen available** |
| --- | --- | --- |
|  | **(N=536)** | **(N=613)** |
| **Clark score** | |  |
| Absent | 129 (24.1%) | 322 (52.5%) |
| Non-brisk | 323 (60.3%) | 229 (37.4%) |
| Brisk | 84 (15.7%) | 62 (10.1%) |
| **MIA score** |  |  |
| 0 | 129 (24.1%) | 322 (52.5%) |
| 1 | 246 (45.9%) | 193 (31.5%) |
| 2 | 124 (23.1%) | 73 (11.9%) |
| 3 | 37 (6.9%) | 25 (4.1%) |
| **Stromal score** | |  |
| Median [IQI] | 10 [5- 30] | 5 [0-20] |
| Missing | 21 | 94 |
|  |  |  |

Supplementary table 5. Included versus excluded patients in the cohort with primary specimen available (n = 536) and the cohort with pre-treatment metastatic specimen available (n= 613). Abbreviations: IQI, interquartile interval; WHO, World Health Organization; LDH; Lactate dehydrogenase; ULN, upper limit of normal.

|  | **Patients with primary specimen available** | | **Patients with pre-treatment metastatic specimen available** | |  |
| --- | --- | --- | --- | --- | --- |
|  | **Excluded** | **Included** | **Excluded** | **Included** | **Total** |
|  | **(N=810)** | **(N=536)** | **(N=733)** | **(N=613)** | **(N=1346)** |
| **Age (years)** |  |  |  |  |  |
| Median [IQI] | 67 [57-75] | 68 [58- 75] | 68 [57-76] | 66.0 [57-74] | 68 [57-75] |
| **Sex** |  |  |  |  |  |
| Female | 329 (40.6%) | 186 (34.7%) | 308 (42.0%) | 207 (33.8%) | 515 (38.3%) |
| Male | 481 (59.4%) | 350 (65.3%) | 425 (58.0%) | 406 (66.2%) | 831 (61.7%) |
| **WHO Performance status** | |  |  |  |  |
| WHO 0 | 388 (50.5%) | 243 (47.2%) | 367 (52.6%) | 264 (45.1%) | 631 (49.1%) |
| WHO 1 | 313 (40.7%) | 227 (44.1%) | 262 (37.5%) | 278 (47.4%) | 540 (42.1%) |
| WHO 2-4 | 68 (8.8%) | 45 (8.7%) | 69 (9.9%) | 44 (7.5%) | 113 (8.8%) |
| Missing | 41 | 21 | 35 | 27 | 62 |
| **Stage of disease** | |  |  |  |  |
| Unresectable IIIC | 75 (9.7%) | 39 (7.6%) | 70 (9.9%) | 44 (7.5%) | 114 (8.9%) |
| M1a | 67 (8.7%) | 32 (6.2%) | 62 (8.8%) | 37 (6.3%) | 99 (7.7%) |
| M1b | 103 (13.3%) | 76 (14.8%) | 101 (14.3%) | 78 (13.4%) | 179 (13.9%) |
| M1c | 317 (41.0%) | 248 (48.3%) | 300 (42.6%) | 265 (45.5%) | 565 (43.9%) |
| M1d | 212 (27.4%) | 118 (23.0%) | 171 (24.3%) | 159 (27.3%) | 330 (25.6%) |
| Missing | 36 | 23 | 29 | 30 | 59 (4.4%) |
| **BRAF V600 Mutation** | |  |  |  |  |
| Wildtype | 483 (67.0%) | 354 (74.1%) | 454 (69.4%) | 383 (70.3%) | 837 (69.8%) |
| Mutant | 238 (33.0%) | 124 (25.9%) | 200 (30.6%) | 162 (29.7%) | 362 (30.2%) |
| Missing | 89 | 58 | 79 | 68 | 147 (10.9%) |
| **LDH levels** |  |  |  |  |  |
| Not elevated | 539 (67.6%) | 343 (64.6%) | 492 (68.0%) | 390 (64.5%) | 882 (66.4%) |
| 1-2x ULN | 205 (25.7%) | 140 (26.4%) | 178 (24.6%) | 167 (27.6%) | 345 (26.0%) |
| >2x ULN | 53 (6.6%) | 48 (9.0%) | 53 (7.3%) | 48 (7.9%) | 101 (7.6%) |
| Missing | 13 | 5 | 10 | 8 | 18 |
| **Type of systemic therapy** | |  |  |  |  |
| Anti-PD1 | 529 (65.3%) | 346 (64.6%) | 493 (67.3%) | 382 (62.3%) | 875 (65.0%) |
| Ipilimumab & Nivolumab | 281 | 190 | 240 | 231 | 471 |

Supplementary table 6.
Quasi-Poisson Regression Analysis of the Relationship Between (TIL) Scoring and the Number of Organs with Grade ≥3 Immune-Related Adverse Events (irAEs). Both primary and metastatic samples were included in the analysis. No variable achieved statistical significance, indicating that there is no statistical relationship between the Clark score and the count of toxicity events.

| **Primary samples** | Variable | IRR | 95% CI | p-value |
| --- | --- | --- | --- | --- |
|  | Non-brisk vs absent | 1.28 | 0.86 – 1.97 | 0.24 |
|  | brisk vs absent | 1.10 | 0.66 – 1.92 | 0.73 |
| **Metastatic samples** | **Variable** | **IRR** | **95% CI** | **p-value** |
|  | Non-brisk vs absent | 0.92 | 0.68 – 1.25 | 0.61 |
|  | brisk vs absent | 0.73 | 0.41 – 1.22 | 0.26 |

Supplementary table 7.

Occurrence of grade 3 or higher colitis in patients with primary and metastasis specimen available, and univariable logistic regression analysis of TIL presence (absent versus present) and the development of grade 3 or higher colitis in ICI-treated advanced melanoma patients.

| Occurence of grade 3 or higher colitis in patients with primary specimen available | | 7% | | |
| --- | --- | --- | --- | --- |
| TILs scored in primary melanoma specimen | | | | |
|  | Univariable analysis | | | |
| Presence of TILs | OR | | 95% CI | p-value |
| Absent | - | | | |
| Present | 1.16 | | 0.54-2.78 | 0.7 |
| Occurence of grade 3 or higher colitis in patients with primary specimen available | | 8% | | |
| TILs scored in pre-treatment metastasis specimen | | | | |
| Presence of TILs | OR | | 95% CI | p-value |
| Absent | - | | | |
| Present | 0.89 | | 0.49-1.60 | 0.7 |

Supplementary figure 1.

Supplementary figure 2. Stacked bar chart comparing the occurrence of BRAFV600 and NRAS mutational status in patients categorized by their TILs score (absent, non-brisk, or brisk). The comparison is shown for BRAFV600 mutational status in patients with primary specimens available (A, p=0.059) and pre-treatment metastatic specimens available (B, p=0.66), and for NRAS mutational status in patients with primary samples (C, p=0.07) or metastatic samples (D, p=0.41).


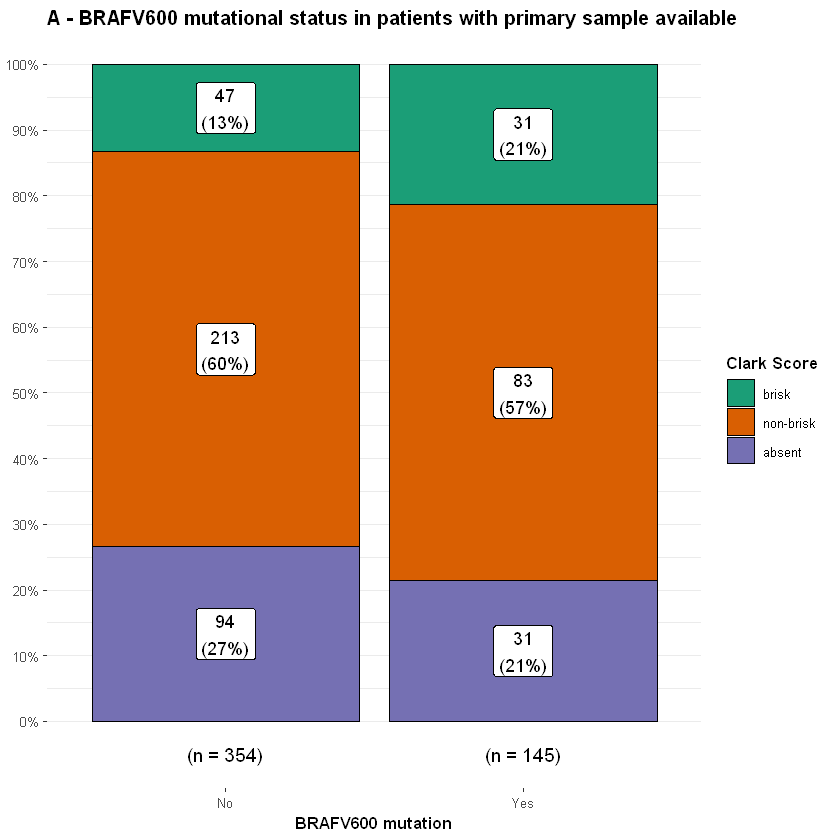

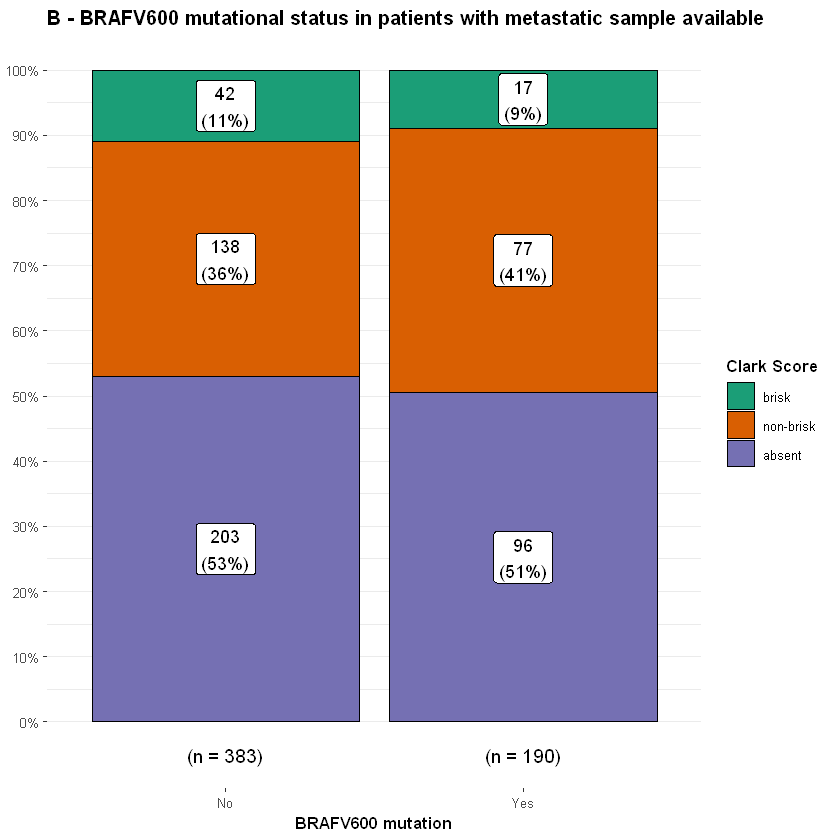

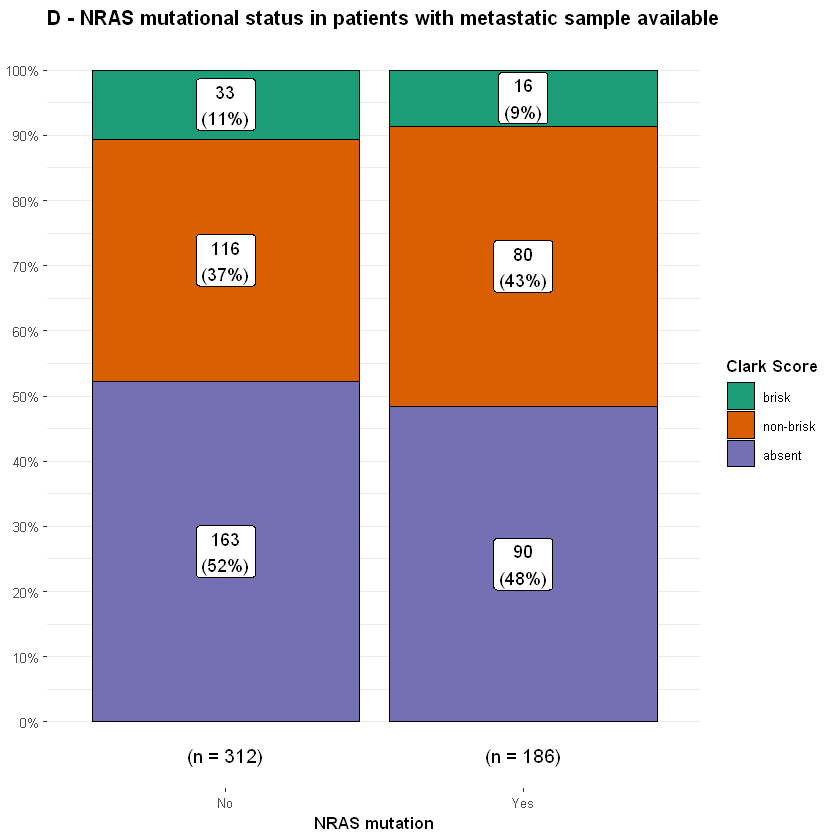

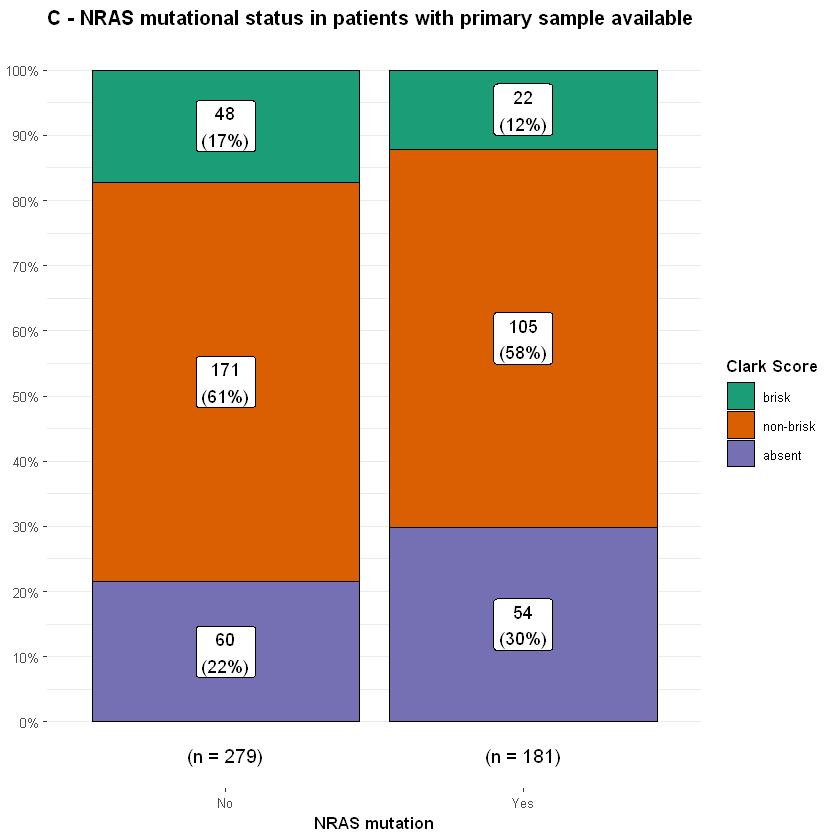


Supplementary figure 3. Stacked barcharts and boxplots of comparison of occurrence of grade ≥3 irAEs in patients categorized by their TILs score. (A+B) Patients with primary specimen available scored according to the ‘MIA’ score (A, p=0.56) and the ‘stromal’ score (B, p=0.86). (C+D) Patients with pre-treatment metastatic specimen available according to the ‘MIA’ score (C, p=0.93) and the ‘stromal’ score (D, p=0.57)

Supplementary figure 4. Stacked barchart of comparison of occurrence of grade ≥3 irAEs in patients categorized by their TILs score (absent, non-brisk or brisk) in treatment subgroups. (A+B) Anti-PD-1 treated patients with primary specimen available (A, p=0.41) and with pre-treatment metastatic specimen available (B, p=0.66). (C+D) Ipilimumab & Nivolumab treated patients with primary specimen available (C, p=0.81) and with pre-treatment metastatic specimen available (D, p=0.82).

Supplementary figure 5. Flowchart of the studied population in the time to toxicity analysis

Supplemental references

1. Clark, W. H. *et al.* Model predicting survival in stage I melanoma based on tumor progression. *J Natl Cancer Inst* **81**, 1893–1904 (1989).

2. Clemente, C. G. *et al.* Prognostic value of tumor infiltrating lymphocytes in the vertical growth phase of primary cutaneous melanoma. *Cancer* **77**, 1303–1310 (1996).

3. Azimi, F. *et al.* Tumor-Infiltrating Lymphocyte Grade Is an Independent Predictor of Sentinel Lymph Node Status and Survival in Patients With Cutaneous Melanoma. *JCO* **30**, 2678–2683 (2012).

4. Hendry, S. *et al.* Assessing tumor infiltrating lymphocytes in solid tumors: a practical review for pathologists and proposal for a standardized method from the International Immuno-Oncology Biomarkers Working Group. *Adv Anat Pathol* **24**, 311–335 (2017).
