# Supplementary material for: Tumor-infiltrating lymphocytes and immune-related adverse events in advanced melanoma
Source: Immunooncol Technol. 2024 Jun 12;24:100714. doi: 10.1016/j.iotech.2024.100714 (PMC11262179; doi:10.1016/j.iotech.2024.100714)
Supplement: Supplementary material [file mmc2.docx]

Supplementary Figure 1A-C. Visual representation of TIL score in pre-treatment metastatic sample. (A) Brisk TILs in lymph node metastasis, (B) non-brisk TILs in lymph node metastasis, (C) absent TILs in cutaneous metastasis.
